# Fabrication of nanoscale Ga balls *via* a Coulomb explosion of microscale silica-covered Ga balls by TEM electron-beam irradiation

**DOI:** 10.1038/srep11313

**Published:** 2015-06-23

**Authors:** Ying Chen, Yanli Huang, Nishuang Liu, Jun Su, Luying Li, Yihua Gao

**Affiliations:** 1 Center for Nanoscale Characterization and Devices (CNCD), Wuhan National Laboratory for Optoelectronics (WNLO)-School of Physics, Huazhong University of Science and Technology (HUST), Luoyu Road 1037, Wuhan 430074, P. R. China

## Abstract

Nanoscale Ga particles down to 5 nm were fabricated by an explosion *via* an *in situ* electron-beam irradiation on microscale silica-covered Ga balls in a transmission electron microscope. The explosion is confirmed to be a Coulomb explosion because it occurs on the surface rather than in the whole body of the insulating silica-covered Ga micro–balls, and on the pure Ga nano-balls on the edge of carbon film. The ejected particles in the explosion increase their sizes with increasing irradiation time until the stop of the explosion, but decrease their sizes with increasing distance from the original ball. The Coulomb explosion suggests a novel method to fabricate nanoscale metal particles with low melting point.

Gallium, one of the liquid metals in ambient temperature, has attracted considerable interest due to its unique properties (low melting point (29.8 °C), rich phase diagram, etc.)^1^ and wide applications (surface-enhanced Raman scattering^2^,^3^, optical memory element^4^,^5^ and self-healing anode in high performance Li-ion batteries^6^,^7^). Furthermore, Ga nanoparticles are also widely used as catalyst, e.g. Ga filling nanotube nanothermometers^8^,^9^,^10^, highly aligned silica nanowires^11^, and SnO_2_ nanowires and nanobelts^12^. Unlike other metal catalysts (Au, Ag, Pt, etc.) require high temperature during catalytic process of vapor-liquid-solid (VLS) growth^13^,^14^,^15^, Wang *et al*. reported the fabrication of ZnO nanotubes at relatively low temperature (80 °C) using Ga as catalyst^16^. Moreover, Low melting point also makes Ga nanoparticles as a potential low temperature catalyst for solution-liquid-solid (SLS) growth of nanostructures^17^. Nanowires produced by SLS mechanism have advantages like good dispersibility and small diameters in the range of ca. 4–12 nm. Low temperature catalysts enable us to synthesize nanostructures on various substrates which are not resistant to high temperature (eg. flexible or transparent organic films), and have enormous potential applications in the design of flexible or transparent electronics.

Nowadays, some strategies, thermal evaporation^1^, sonication^6^, molecular-beam epitaxy^2^,^18^, are commonly used for the fabrication of Ga nanoparticles. Arbiol *et al*. synthesized colloidal Ga nanoparticles by chemical liquid deposition (CLD) at 77 K^19^. Recently, Kovalenko *et al*. reported a facile colloidal synthesis of gallium nanoparticles with the mean size tunable in the range of 12–46 nm using conventional solution-phase chemistry^20^. Besides, transmission electron microscope (TEM) is proved to be a powerful tool to engineer and modify nanostructures in recent decades. Herein, we report the fabrication of Ga nanoparticles with the diameters in the range of 5–500 nm in an *in situ* TEM by using electron beam to irradiate the microscale silica-covered Ga balls, and suggest a charging explosion method for fabricating nanoscale metal particles. The particle size can be controlled by the irradiation time, and we can observe the morphology momentarily. Golberg *et al*. reported an *in situ* Coulomb explosion phenomenon by electron beam irradiation in a TEM^21^, and Gao *et al*. reported an abnormally large and fast expansion of Ga in insulating silica-shelled tube by electron beam irradiation in TEM, and suggested that this expansion was a Coulomb expansion^22^ due to Coulomb repulsion. Here we keep our mind on the explosion characteristic of materials under electron beam irradiation. Since a macroscopic thermal explosion usually occurs in the whole body and a Coulombic explosion occurs on surface with charges, the characteristic of regions where the explosion occurs under electron irradiation should be the focus of study. If the explosion happens on the surface, the conclusion of Coulombic characteristic of the explosion is more persuadable, and charging is the essential factor for the electron beam induced explosion.

A model is proposed as shown in Fig. 1(a) to illustrate the Coulomb explosion of a microscale silica-covered Ga ball. *R*_*0*_ is the radius of the Ga ball, and *d* is the thickness of the silica shell. When the electron beam irradiates the Ga ball, the electrons inside the Ga ball will obtain kinetic energy by absorbing energy from the incident electrons, and excited out from the Ga ball when the electron energy is higher than its work function^23^, i.e. a secondary electron emission occurs. The Ga atoms will restore electrostatic equilibrium if the sample is connected outside through good electrical conductive channel. In the current study, there is a shielding effect because the silica shell has a bad electrical conductivity, which can reduce the degree of electron back scattering^24^, improve the inelastic scattering of electrons in the Ga microball, and the accumulation of positive Ga ions on the surface of Ga ball. As shown in Fig. 1(a), the positive charges on the surface will generate a strong Coulomb repulsion.

An explosion is usually due to drastic expansion originated from big inner pressure generated during extremely short time period. In a silica-shelled Ga ball irradiated by electron beam, the most possible origin of the big inner pressure is Coulomb repulsion, as analyzed below. The thermal conductivities of Ga^25^ and silica^26^ are 30.6 W/ m·K and ~1.38 W/m·K, respectively. The temperature rise due to electron beam heating in a TEM is very small for micro/nanoscale particles, and the heat flow is too fast to maintain high temperature in silica-shelled Ga balls^27^. Therefore, the pressure increase due to the heating effect of electron beam irradiation can be neglected. Moreover, the electrical resistance of insulating silica (~10^10^ Ω·cm)^26^ and Ga (2.58 × 10^−5^ Ω·cm)^25^ has a big ratio of ~3.87 × 10^14^, which is much bigger than heat resistance ratio ~22 of silica and Ga, and suggests that charge accumulation on the surface is much easier than heat accumulation in the body. In addition, a macroscopic thermal explosion usually occurs in the whole body, and a Coulombic explosion occurs on the surface since the charges tend to move to the materials surface. Therefore, a conclusion of Coulombic characteristic is more persuadable, and charging is the essential factor for the electron beam induced explosion if an explosion occurs on the surface.

For a silica-shelled Ga ball shown in Fig. 1(a), there are three primary pressures *p*, *p*_1_ and *p*_2_ in the electron beam irradiated silica-shelled Ga balls, where *p* is the electric field stress of electrostatic repulsive force between positive Ga ions (*p* = *σ*^2^/*ε*_0_), *p*_1_ is the ultimate stress of the silica spherical shell, *p*_2_ is the surface tension stress of liquid Ga^22^,^28^. According to the first strength theory, *p*_*1*_ = 2*dσ*_*b*_/*R*_0_. The pressure difference across the fluid interface can be calculated by Young–Laplace equation, *p*_*2*_ = 2*α*/*R*_0_. Here *p* results in the repulsion, *p*_1_ and *p*_2_ prevent the repulsion. We can establish equation (1) to interpret the critical point of explosion based on the above analysis:


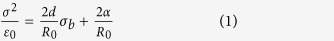


where σ is the surface charge density of the irradiated Ga ball, *σ*_*b*_ ~ 5 × 10^7^ Pa is the tensile stress of silica^26^, *α=*0.881 Jm^2^ is the surface energy of Ga^29^, and *ε*_0_ = 8.85 × 10^−12^ C^2^/Nm^2^. If we assume R_0_ = 2 μm and d = 10 nm. The critical value of *σ* is calculated to be ~3.496 × 10^−3^ C/m^2^, and the explosion will happen when the surface charge density *σ* is bigger than the critical value. The value of 3.496 × 10^−3^ C/m^2^ means that 0.0776% of the total Ga surface atoms will loss 2 electrons per atom (or 0.310 ppm of total Ga atoms in the microball)^22^.

To verify the validity of our analytical result, we also irradiated other pure Ga particles. For a pure Ga particle, there are two possible locations, as shown in Fig. 1(b). Two situations: (i) the Ga ball is grounded well through the C film covering Cu grid, TEM holder, TEM and the Earth. No charge accumulation occurs on the Ga ball surface even a strong electron beam irradiates it. (ii) The Ga ball is dangling on the edge of the C film supported on the Cu grid. The generated positive charges due to electron beam irradiation on a Ga ball cannot be restored immediately by the outside coming electrons from the Earth because the conductive path is not smooth. As the accumulation of positive charges on the surface, an explosion occurs at the critical point. The critical point of explosion can be deduced from (1) since p_1_ for silica is absent, 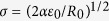. The Coulomb explosion can also happen when a high voltage is applied to the little Ga particles. When the electric field strength is higher than the critical field strength (15 V/nm for Ga) of field evaporation^30^, the Coulomb explosion (ion emission) will happen.

A Ga ball covered by silica shell before irradiation is shown in Fig. 2(a). The radius of the ball reduced gradually from ~2.1 μm to ~1.8 μm with increasing irradiation time, and some small Ga particles were ejected from the surface of the original ball and distributed around the ball as shown in Fig. 2(b–e). The diameter of the ejected Ga particles kept increasing until the explosion stopped. It is a Coulomb explosion from the ball surface due to positive charge accumulation on the Ga ball surface. According to the above estimation, the explosion can occur at the situation that only more than 0.310 ppm of total Ga atoms in the microball losing 2 electrons per atom since the radius is ~2 μm. The EDS spectra in Fig. 2(f) are collected from the nanoscale Ga ball covered by silica in Fig. 2(a) before explosion. This result indicates that the original Ga ball contains silicon and oxygen in the form of Ga and Si oxides on the surface of the Ga ball.

Figure 3 shows another group of Ga particles exploded from the Ga ball in Fig. 2(a). Four Ga particles marked by black arrows (Fig. 3(a)) are dangling on the edge of the carbon film. As shown in Fig. 3(b), the four Ga particles after electron beam irradiation for 2890 s decrease their sizes from ~14 nm to ~11 nm, which should be due to the Coulomb explosion in the 2^nd^ situation in Fig. 1(b): since the Ga ball is dangling on the C film, the generated positive charges due to electron beam irradiation cannot be restored and at last an explosion occurs. According to the above estimation, the critical value of *σ* is calculated to be ~3.605 × 10^−2^ C/m^2^ since the radius is ~12 nm, which means that 0.053% of total Ga atoms in the nanoball will lose 2 electrons per atom. For comparison, some nanoparticles without electron irradiation do not change their sizes, as shown in the circled region in Fig. 3(a,b).

Some Ga particles ejected from the original silica-shelled Ga ball (in Fig. 2(a)) are irradiated again by electron beam, as shown in Fig. 4. The Ga particles shown in Fig. 4(b) were irradiated for 2904 s, but their sizes almost remained (Fig. 4(a)). We can understand the phenomenon as in the 1^st^ situation in Fig. 1(b): when the electron beam irradiates the Ga particles in the centre of carbon film, the positive charges will be neutralized by coming electrons immediately. Thus, no explosion would happen to those Ga particles.

It is found that the distribution of the exploded particles complies with a law during the explosion of microscale Ga balls covered by silica: the size of the exploded particles decreases almost linearly with increasing distance from the center ball, as demonstrated in a case at the moment of 1158 s in Fig. 5(a,b) shows that the diameters of exploded particles increase with increasing irradiation time at different distances (2.97 μm, 3.39 μm, 3.75 μm, 3.88 μm, 4.47 μm, 4.53 μm) from the center ball. The exploded Ga particles nearer to the center ball are bigger, and the increasing rate of the ball size decreases with increasing irradiation time. Figure 6(a) shows a randomly selected area of carbon film covered with Ga nanoparticles. The mean size of Ga nanoparticles is ~18 nm, and with a standard deviation of ~8 nm. At last, we studied the crystallization of Ga nanoparticles at low temperature. The Ga nanoparticles were successfully crystallized when cooling down to 90 K, and we can see clear lattice fringes in Fig. 6(b). Several 5–10 nm Ga nanoparticles don’t show evidence of crystallization (Fig. 6(c)), and they may still stay in liquid state at 90 K due to undercooling of liquid Ga nanoparticles^31^. The exploded particles have diameters in the range of 5–500 nm depending on the distances from the center ball. Thus, we can obtain Ga nanoparticles with different diameters through the explosion method.

In conclusion, a new method of obtaining nanoscale materials without reuniting or transferring is realized by *in situ* TEM technique. It is observed that Coulomb explosion taken place on the surface of microscale Ga balls covered by insulating silica, and nanoscale Ga balls dangling on the edge of C film covering Cu grid under electron beam irradiation in TEM. The size of the exploded particles decreases almost linearly with increasing distance from the center ball, and the increasing rate of the ball size decreases with increasing irradiation time. The Coulomb explosion suggests a novel method to fabricate nanoscale metal particles with low melting point.

## Methods

### Growth of the Ga balls covered by silica shell

The growth procedure for the silica covered Ga balls was conducted in a vertical induction furnace consisting of a fused-quartz tube and an induction-heated cylinder, which has been described elsewhere^8^,^9^,^21^,^32^. Briefly, silicon monoxide and gallium oxide were mixed at an optimized ratio, loaded into a graphite crucible and placed in the central area of the induction heater. During the reaction, protecting nitrogen with flowing rates of 500 and 300 sccm were introduced through the inlets at the top and bottom, respectively. The furnace was heated to 1350 °C. After reaction for 1 h, grey products were collected from the internal wall of the graphite crucible. The chemical reactions are as follows:


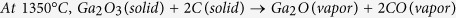



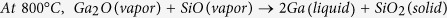


### TEM and EDS characterization

The synthesized product was characterized and irradiated using a transmission electron microscope (Tecnai-G^2^ 20U-TWIN) equipped with energy dispersive X-ray spectroscopy (EDS). The specimens were cooled by a temperature controlled liquid-nitrogen holder (Titan G^2^ 60–300, Gatan’s Model 613 single tilt liquid nitrogen cooling holder).

## Additional Information

**How to cite this article**: Chen, Y. *et al*. Fabrication of nanoscale Ga balls *via* a Coulomb explosion of microscale silica-covered Ga balls by TEM electron-beam irradiation. *Sci. Rep.* **5**, 11313; doi: 10.1038/srep11313 (2015).

## Figures and Tables

**Figure 1:**
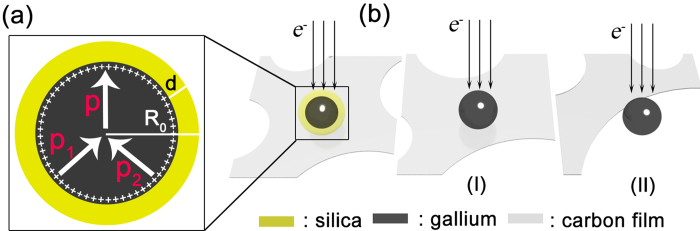
Three situations of a Ga ball on carbon film. (**a**) The Ga ball is covered by silica shell. (**b**) A pure Ga ball is located in the centre of the carbon film (I) or attached to the edge of carbon film (II).

**Figure 2:**
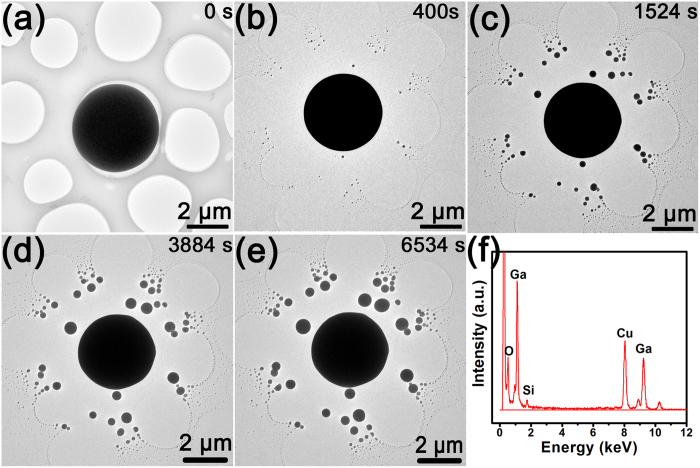
The morphologies and chemical information of the silica covered microscale Ga ball before and after irradiation. (**a**) TEM image of Ga ball before irradiation. (**b**–**e**) After irradiation of ~400 s, 1524 s, 3884 s and 6534 s, respectively. (**f**) The EDS spectra of the silica-shelled microscale Ga ball before irradiation.

**Figure 3:**
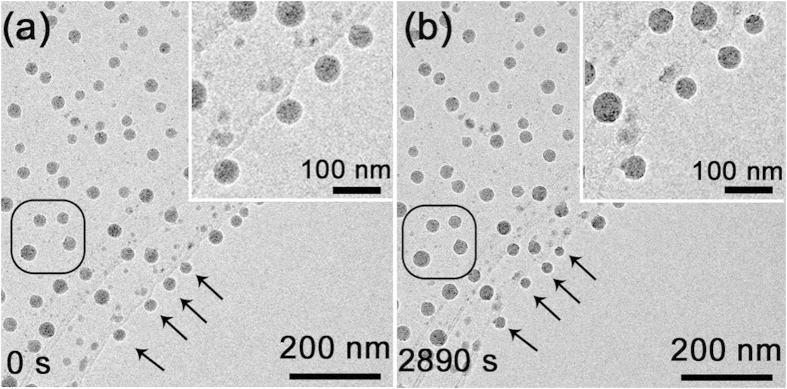
The size change of the Ga particles exploded from the original Ga ball (Fig. 2(a)) covered by silica. The four Ga particles marked by black arrows before irradiation (**a**) decreased their sizes after electron irradiation for 2890 s (**b**). The insets show the enlarged images of the region marked by arrows.

**Figure 4:**
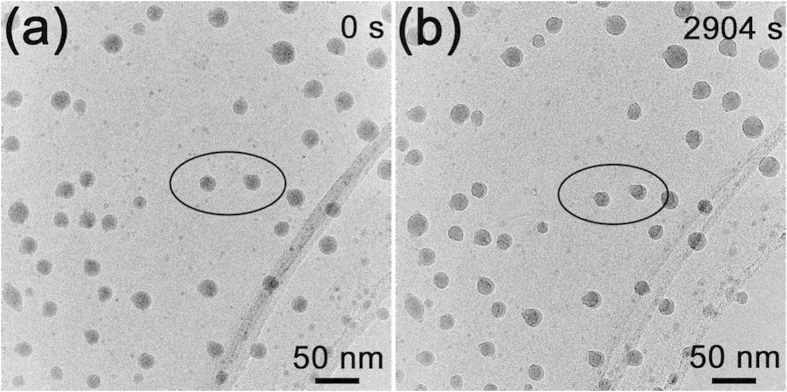
The two situations of the Ga particles exploded from the original Ga ball (Fig. 2(a)) covered by silica before (**a**) and after (**b**) irradiation for 2904 s.

**Figure 5:**
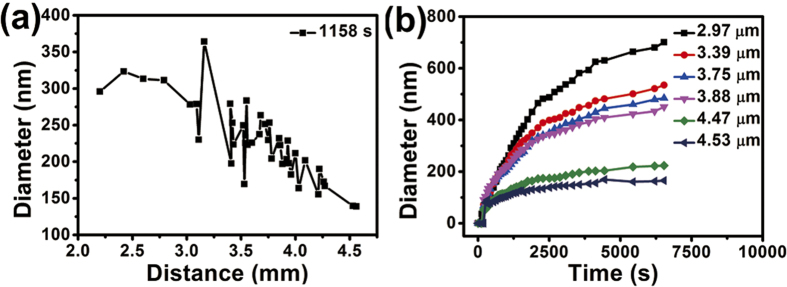
The relations of the diameter of the ejected Ga balls vs the distance from the center ball to the exploded particles, and the diameter of the Ga balls vs explosion time. (**a**) The diameters of the exploded particles decrease with increasing distance at the irradiation time of 1158 s; (**b**) The diameters increase with increasing irradiation time at different distances from the center ball.

**Figure 6:**
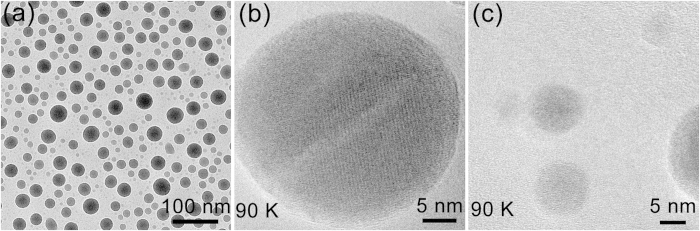
TEM images of the obtained Ga nanoparticles. (**a**) Low-resolution TEM image of Ga nanoparticles at room temperature. (**b**) and (**c**) High-resolution TEM images of Ga nanoparticles with different sizes at 90 K.
